# Supplementary material for: Structures of Atm1 provide insight into [2Fe-2S] cluster export from mitochondria
Source: Nat Commun. 2022 Jul 27;13:4339. doi: 10.1038/s41467-022-32006-8 (PMC9329353; doi:10.1038/s41467-022-32006-8)

Supplementary Information

Structures of Atm1 provide insight into [2Fe-2S] cluster export from mitochondria

Ping Li[1], Amber L. Hendricks[2], Yong Wang[3,4], Rhiza Lyne E. Villones[5], Karin Lindkvist-Petersson[1], Gabriele Meloni[5], J. A. Cowan[2], Kaituo Wang[6#] & Pontus Gourdon[1,6#]

[1] Department of Experimental Medical Science, Lund University, Sölvegatan 19, SE-221 84 Lund, Sweden

[2] Department of Chemistry and Biochemistry, The Ohio State University, 100 West 18th Avenue, Columbus, Ohio 43210, USA

[3] Institute of Quantitative Biology, College of Life Sciences, Zhejiang University, Hangzhou 310027, China

[4] The Provincial International Science and Technology Cooperation Base on Engineering Biology, International Campus of Zhejiang University, Haining 314400, China

[5] Department of Chemistry and Biochemistry, The University of Texas at Dallas, 800 W Campbell Rd., Richardson, TX 75080, USA

[6] Department of Biomedical Sciences, Copenhagen University, Maersk Tower 7-9, Nørre Allé 14, DK-2200 Copenhagen N, Denmark

[#] Corresponding authors; KW (kaituo@sund.ku.dk) & PG (pontus.gourdon@med.lu.se)

Supplementary Figure 1

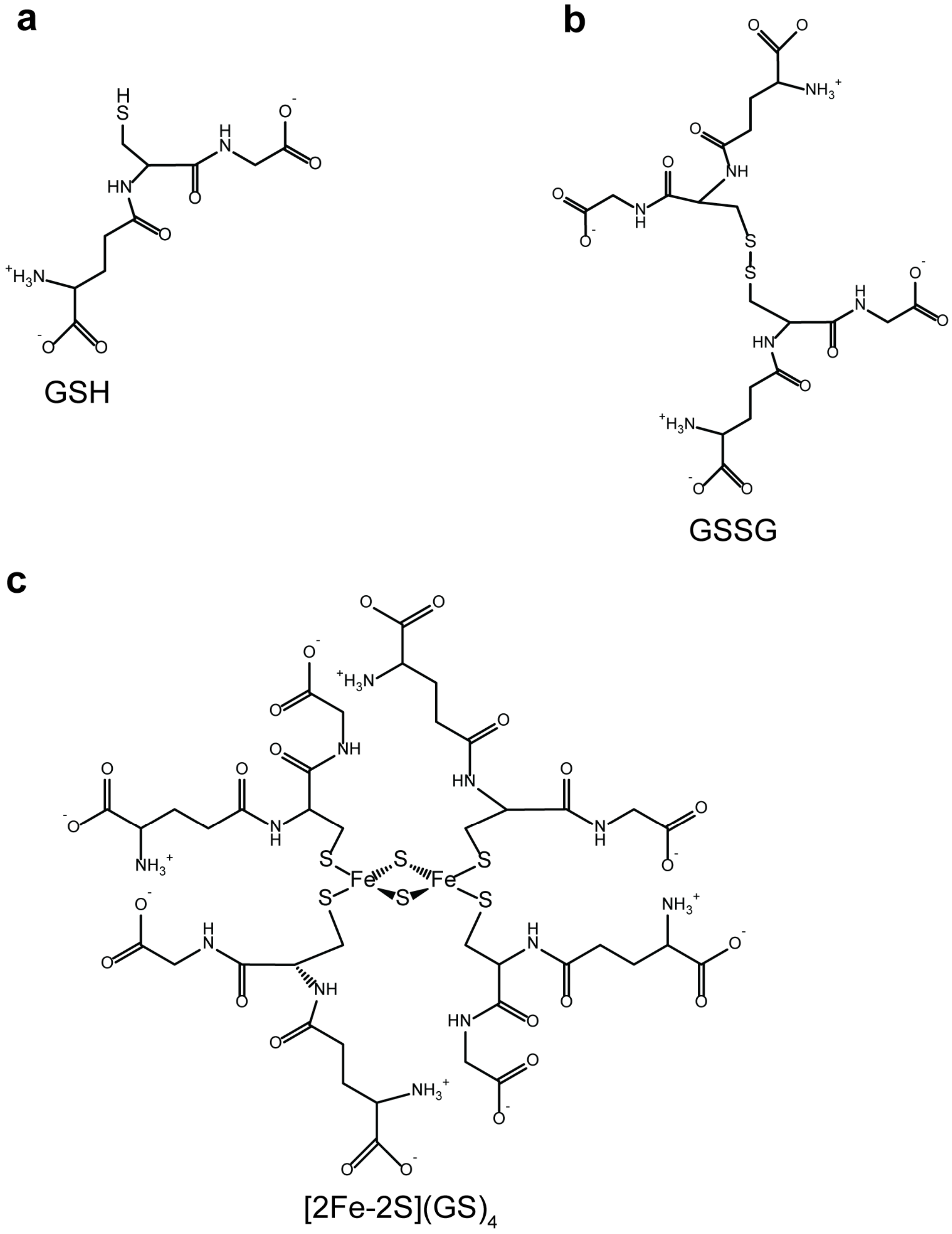

Supplementary Fig. 1: Cartoon structures of molecules that are relevant for the function of eukaryotic Atm1 proteins. a Reduced glutathione (GSH). **b** Oxidized glutathione (GSSG). **c** glutathione bound [2Fe-2S] cluster ([2Fe-2S]$(GS)_4$).

Supplementary Figure 2

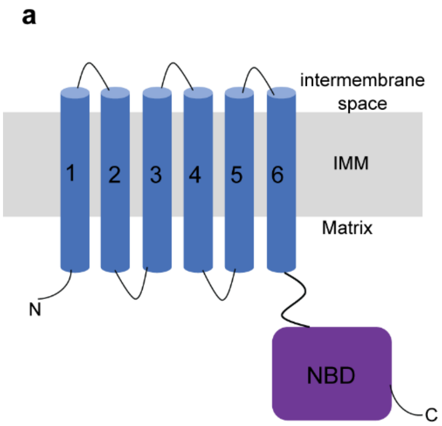

Supplementary Fig. 2: Topology and monomer structure of the Atm1. a Atm1 proteins harbor 6 transmembrane helices forming a transmembrane domain (M1-M6, blue) and a soluble NBD domain (purple). **b** The molecular structure of CtAtm1 shown in rainbow colors. **c** ATPase activity of CtAtm1 in DDM and nanodisc solutions, respectively, without supplementation of cargo to the sample. Data points represent means of 2 replicates of 3 independent biological samples and error bars indicate standard deviation.

Supplementary Figure 3

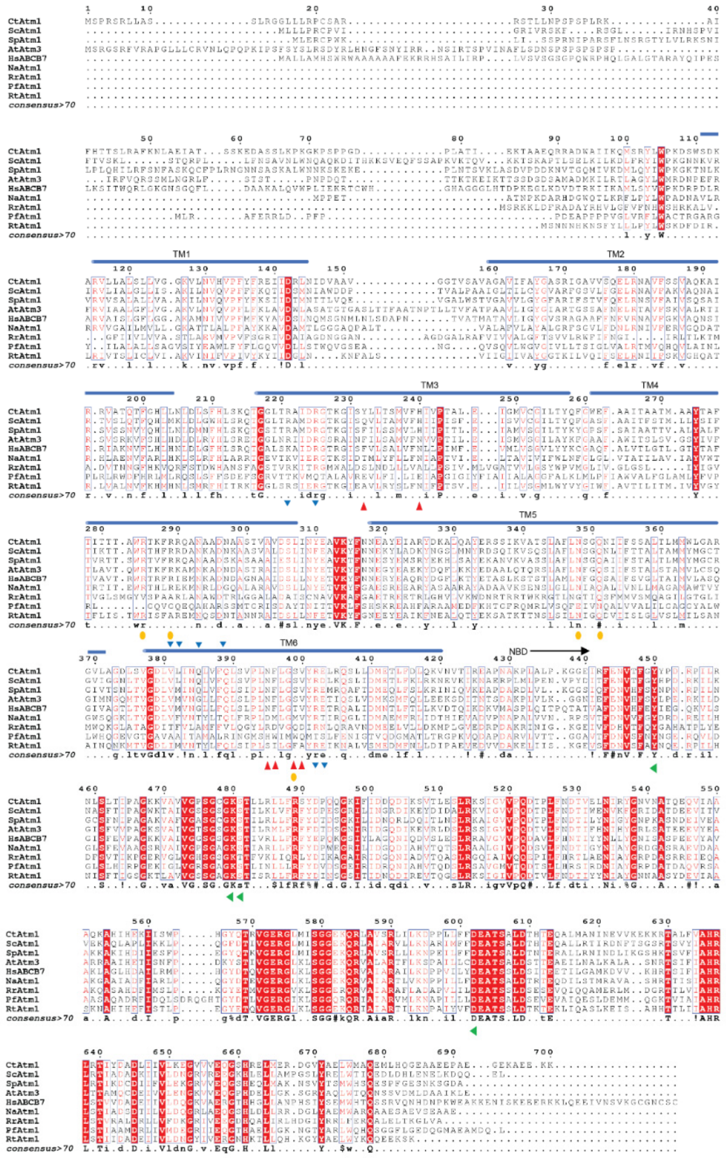

Supplementary Fig. 3: Sequence alignment of eukaryotic and prokaryotic Atm1 proteins. Eukaryotic Atm1: CtAtm1 (Uniport ID G0SBE6), ScAtm1 (P40416), SpAtm1 (O14286), AtAtm3 (Q9LVM1), HsABCB7 (O75027). Prokaryotic Atm1: NaAtm1 (Q2G506), RrAtm1 (A0A083ZLZ4), PfAtm1 (A0A5E7S8C4), RtAtm1(A0A7U0FY61). The gate residues are indicated with red arrowheads. The cluster coordination residues are indicated with blue arrowheads. The GSH coordination residues are indicated with yellow dots. ATP binding residues are indicated with green arrowheads.

Supplementary Figure 4

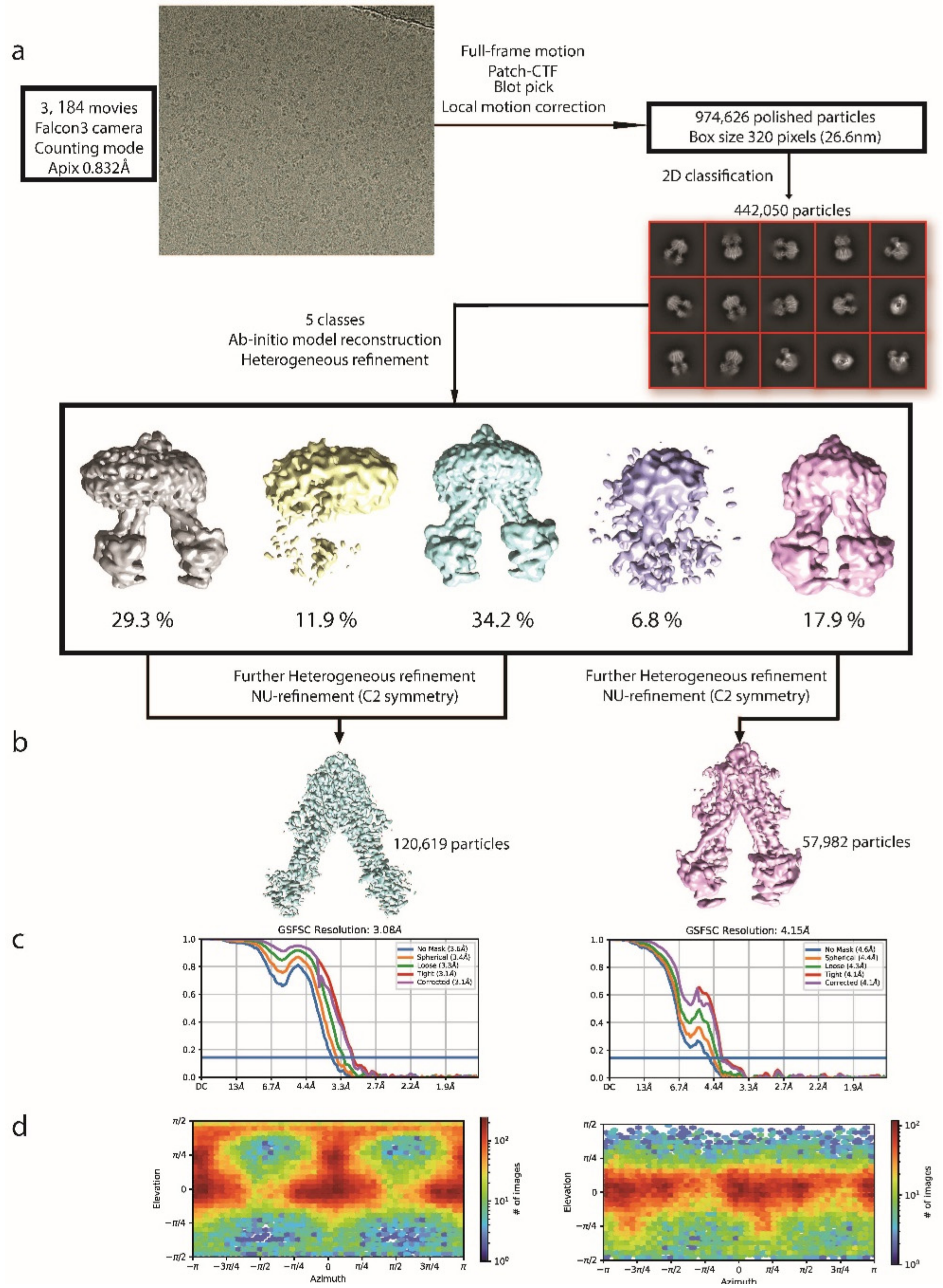

Supplementary Fig. 4: Representative micrograph and Data processing of the CtAtm1$^{inw\text{-}opn}$ state. a Representative micrograph and Data processing flowchart. Please see the Methods section and Table S1 for details. **b** reconstituted 3D cryo-EM maps. **c** Gold standard Fourier shell correlation (FSC) curve of the final maps. **d** Particle orientation distributions in the final 3D reconstruction.

Supplementary Figure 5

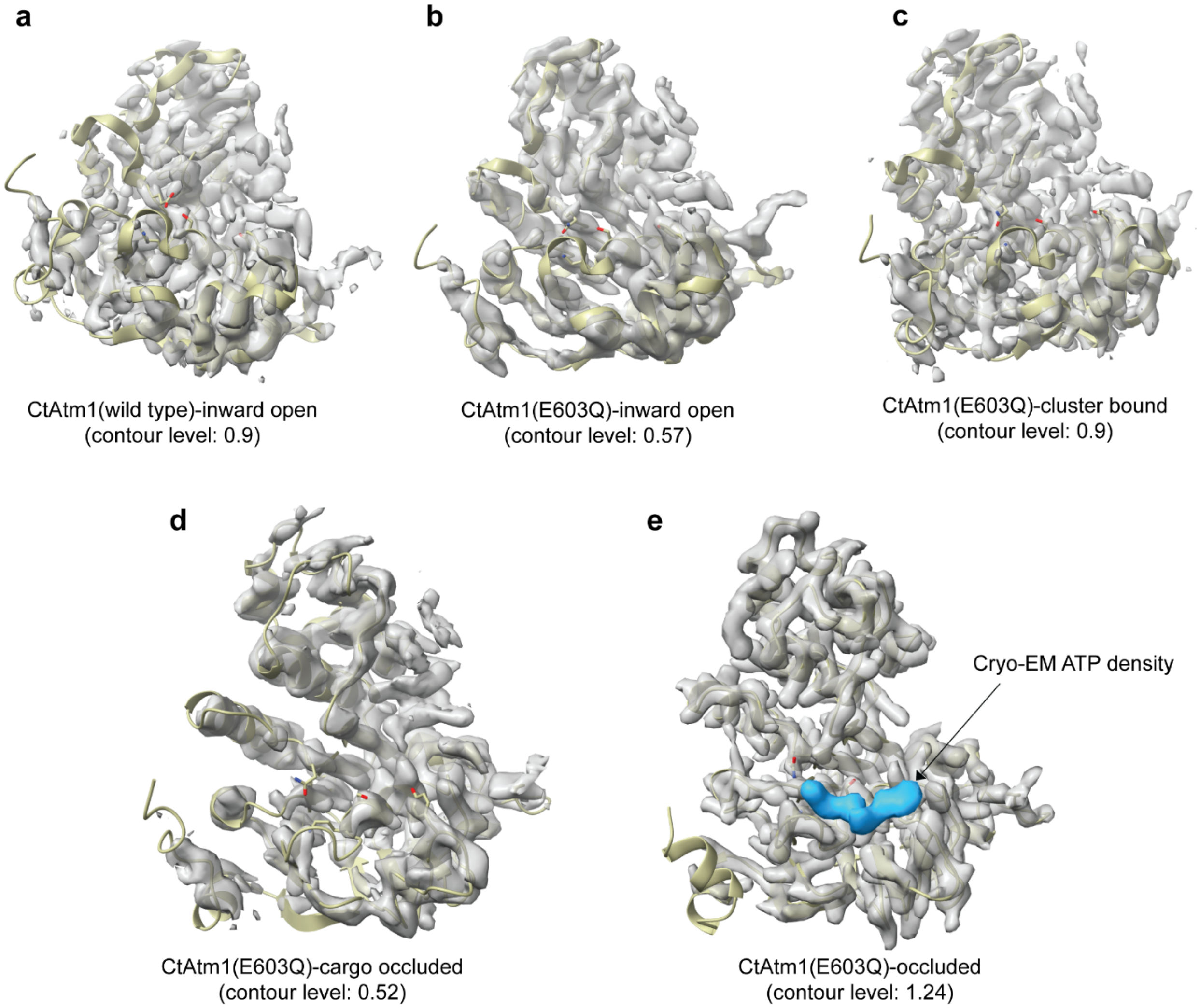

Supplementary Fig. 5: Cryo-EM density quality of the NBDs in different states. The cryo-EM maps are shown in grey surface with 40 % transparency with indicated contour levels. ATP-binding residues Y450, S481, K480, E603/Q603 are shown as sticks for all states. **a** wild-type CtAtm1$^{\text{inw-opn}}$. **b** E603Q CtAtm1$^{\text{inw-opn}}$. **c** CtAtm1$^{\text{inw-opn/cluster}}$. **d** CtAtm1$^{\text{inw-opn-occl}}$. **e** CtAtm1$^{\text{occl/ATP}}$. For the latter, ATP cryo-EM density is shown in blue and is indicated with an arrow.

Supplementary Figure 6

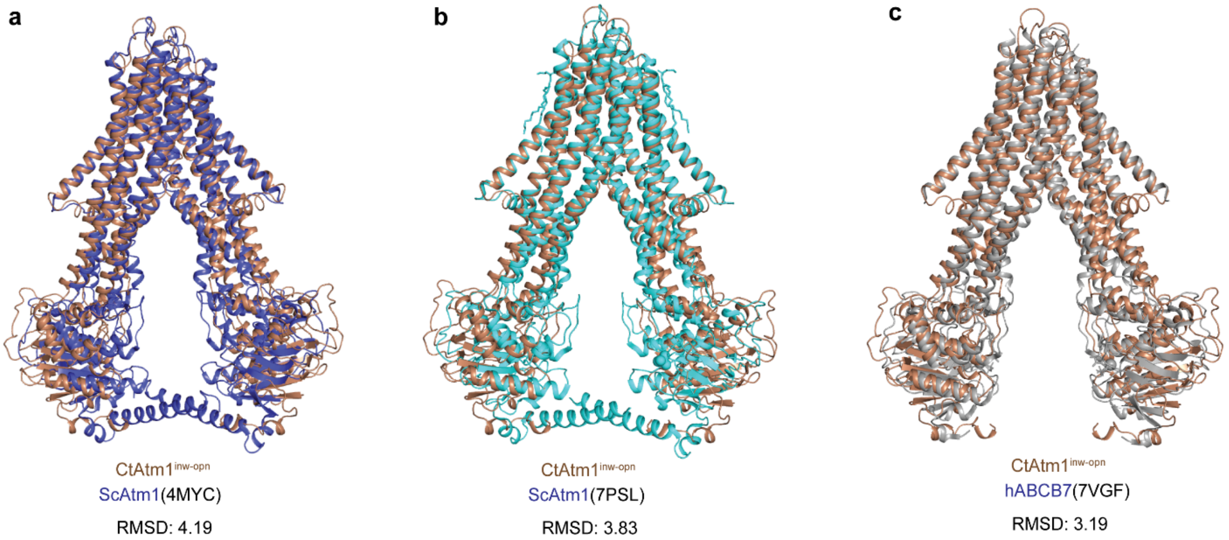

Supplementary Fig. 6: Structural comparison of the inward-facing open state of CtAtm1, CtAtm1$^{inw\text{-}opn}$, with the equivalent conformation of ScAtm1 and hABCB7. a CtAtm1 (brown) is more open than ScAtm1 (PDB-ID 4MYC, dark blue) with an overall RMSD of 4.2 Å. **b** CtAtm1 (brown) is more open than ScAtm1 (PDB-ID 7PSL, cyan) with an overall RMSD of 3.83 Å. **c** CtAtm1 (brown) is relative open than hABCB (PDB-ID 7VGF, grey) with an overall RMSD of 3.19 Å.

Supplementary Figure 7

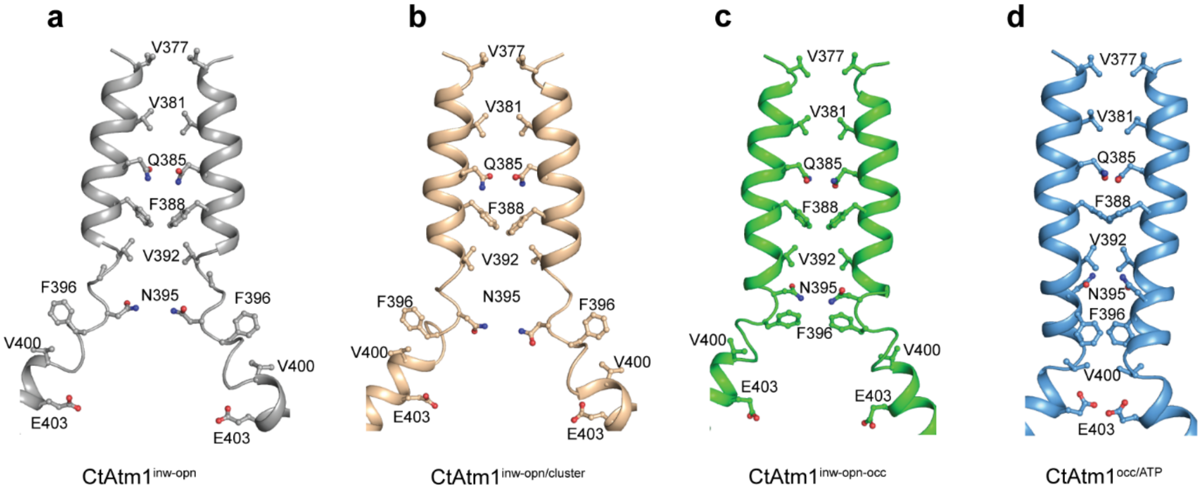

Supplementary Fig. 7: Linking pairs between transmembrane helix TM6 monomers of CtAtm1. Residues are shown as sticks. **a** The inward-facing open state, CtAtm1$^{\text{inw-opn}}$, in grey. **b** The inward-facing open state, CtAtm1$^{\text{inw-opn/cluster}}$, in wheat. **c** The inward-facing partially occluded state, CtAtm1$^{\text{inw-opn-occl}}$, in green. d The occluded state, CtAtm1$^{\text{occl/ATP}}$, in blue.

Supplementary Figure 8

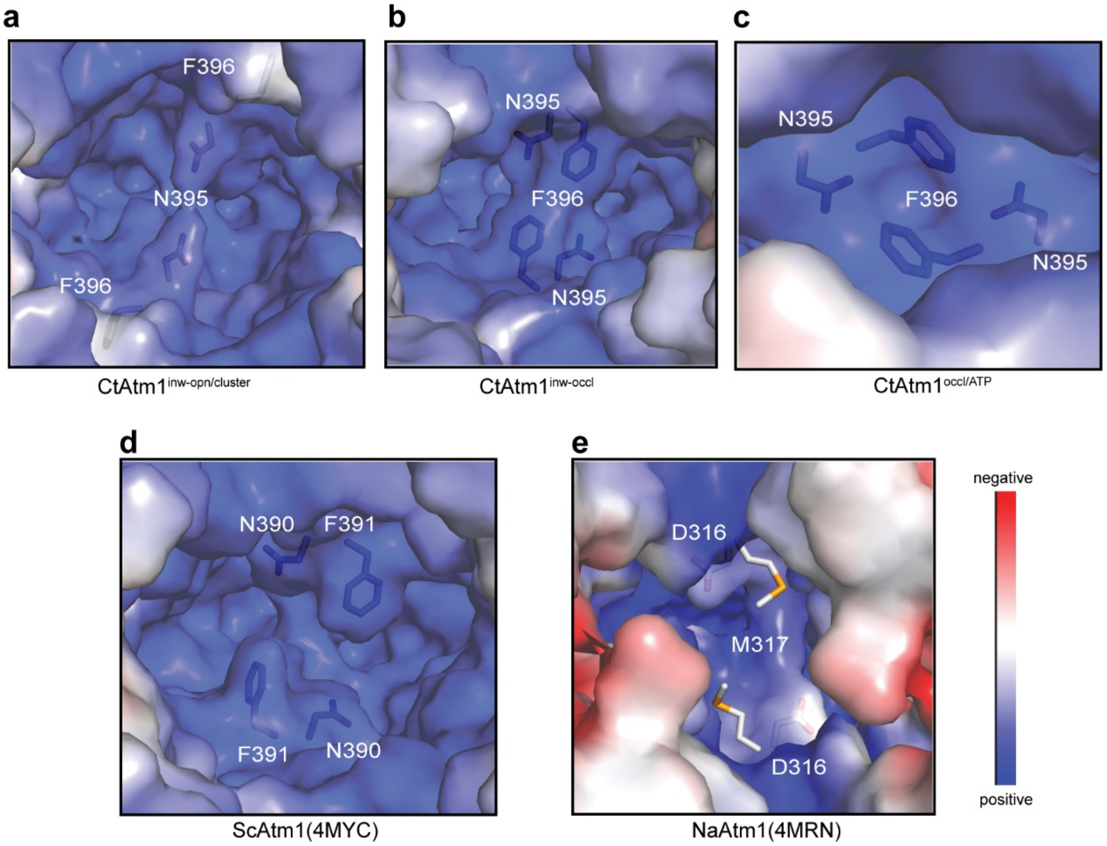

Supplementary Fig. 8: Surface charge of the cavity region in the transmembrane domain. The proteins are cut as shown in Fig. 2b and gate residues are shown as sticks. a The CtAtm1inw-opn/cluster structure. **b** The CtAtm1inw-opn-occl structure. **c** The CtAtm1occl/ATP structure. **d** The cavity of the inward-facing open state of ScAtm1 (PDB-ID 4MYC) is highly positively-charged as in CtAtm1. **e** The cavity of the inward-open structure of NaAtm1 (PDB-ID 4MRN) is less positively-charged pocket. All views are identical with the exception for the CtAtm1occl/ATP structure where the view is closer to the inner gate region, which otherwise is covered by other residues.

Supplementary Figure 9

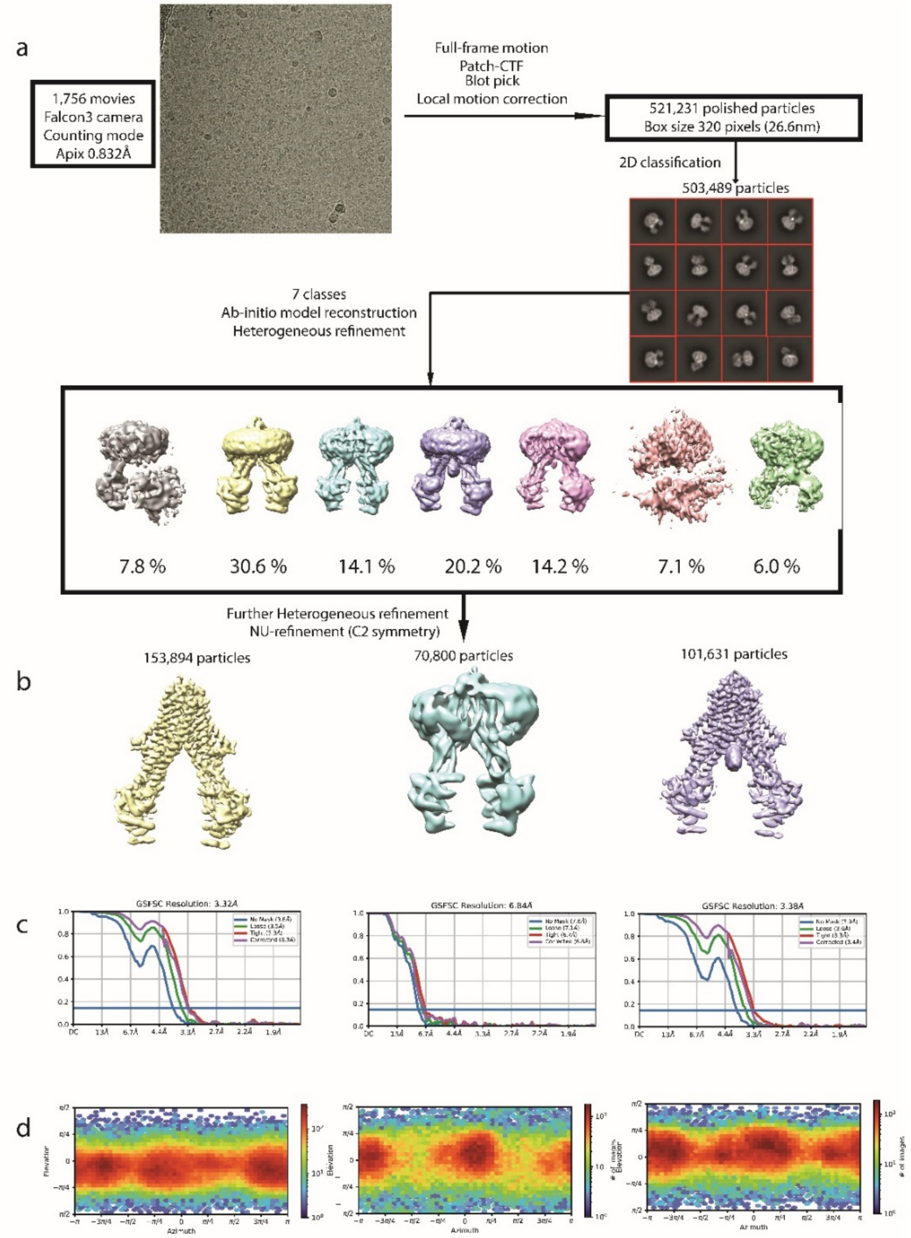

Supplementary Fig. 9: Representative micrograph and data processing of cryo-EM data collected for wild-type CtAtm1 in the presence of the cluster and AMPPNP. a Representative micrograph and data processing flowchart. Please see the Methods section and Table S1 for details. **b** Reconstituted 3D cryo-EM maps with three different states. **c** Gold standard Fourier shell. **d** Particle orientation distributions in the final 3D reconstruction.

Supplementary Figure 10

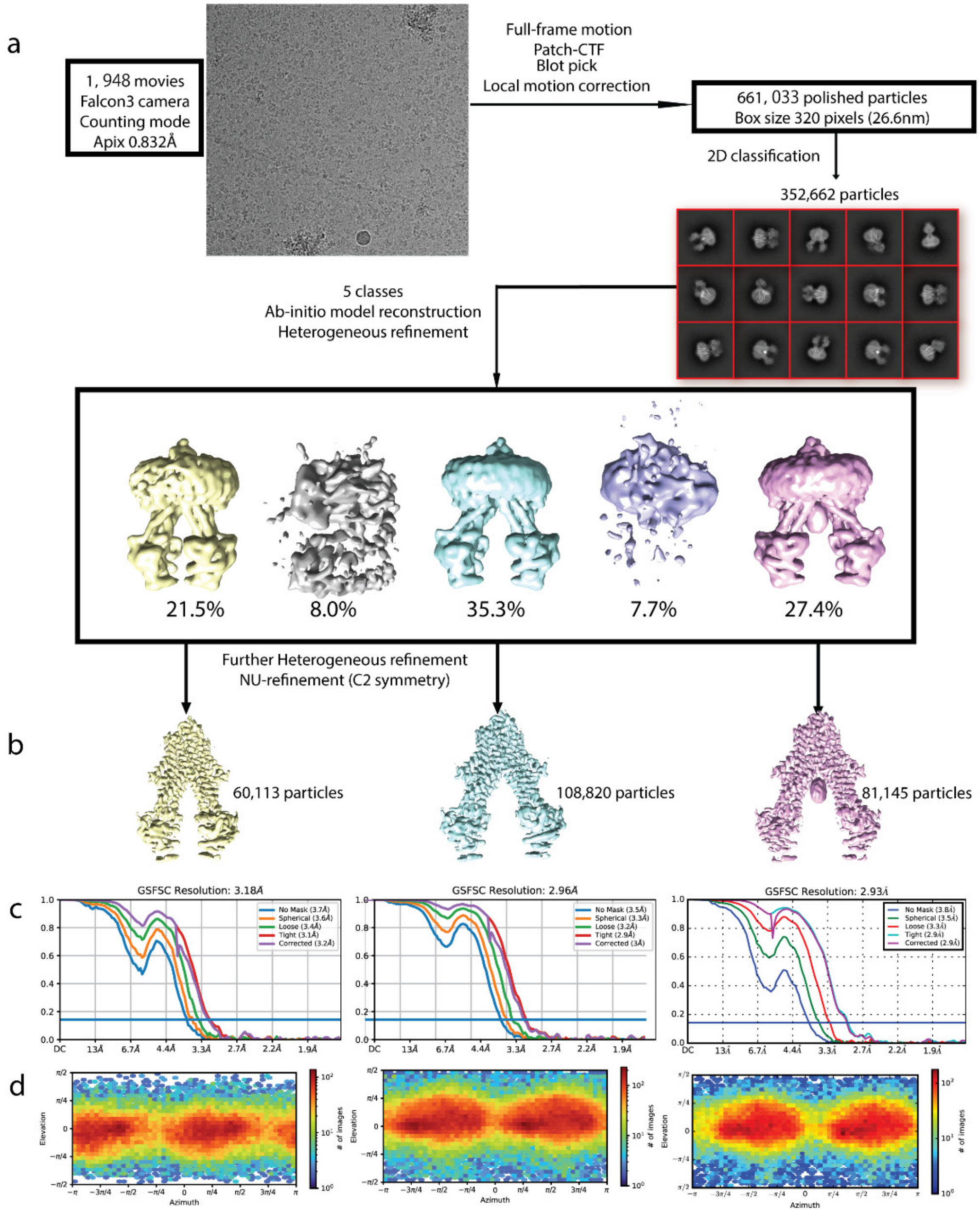

Supplementary Fig. 10: Representative micrograph and data processing of the E603Q CtAtm1$^{inw\text{-}opn}$, CtAtm1$^{inw\text{-}opn/cluster}$, and CtAtm1$^{inw\text{-}opn\text{-}occluded}$ states. a Representative micrograph and data processing flowchart. Please see the Methods section and Table S1 for details. **b** Reconstituted 3D cryo-EM maps with three different states. **c** Gold standard Fourier shell correlation (FSC) curves of the final maps. **d** Particle orientation distributions in the final 3D reconstruction in different states.

Supplementary Figure 11

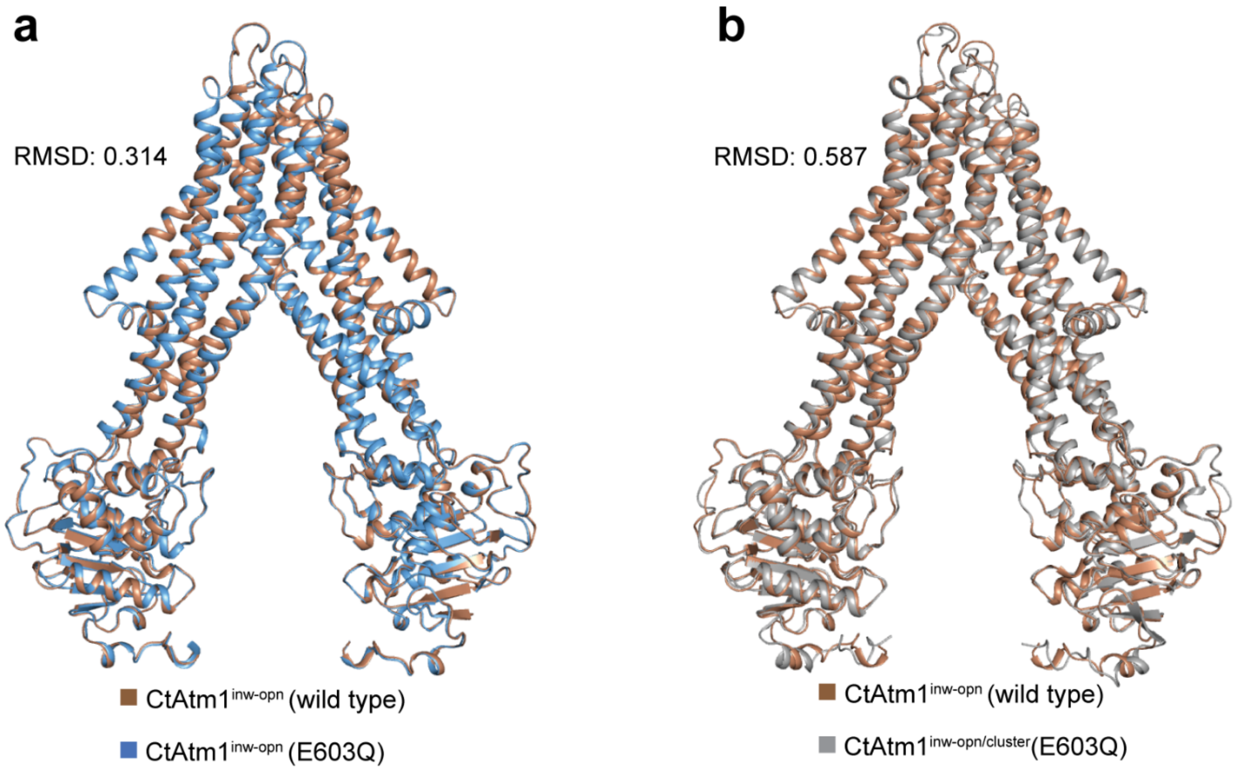

Supplementary Fig. 11: Structural comparison of different inward-facing open CtAtm1 states. The three inward-facing open states are almost indistinguishable overall. **a** Structural alignment of inward-facing open states of wild-type CtAtm1, CtAtm1$^{inw\text{-}opn}$ (brown), with the equivalent of E603Q (blue) with overall RMSD 0.31 Å. **b** Structural alignment of CtAtm1$^{inw\text{-}opn}$ (brown) the cluster bound inward-facing open state, CtAtm1$^{inw\text{-}opn/cluster}$ (grey), with overall RMSD 0.59 Å.

Supplementary Figure 12

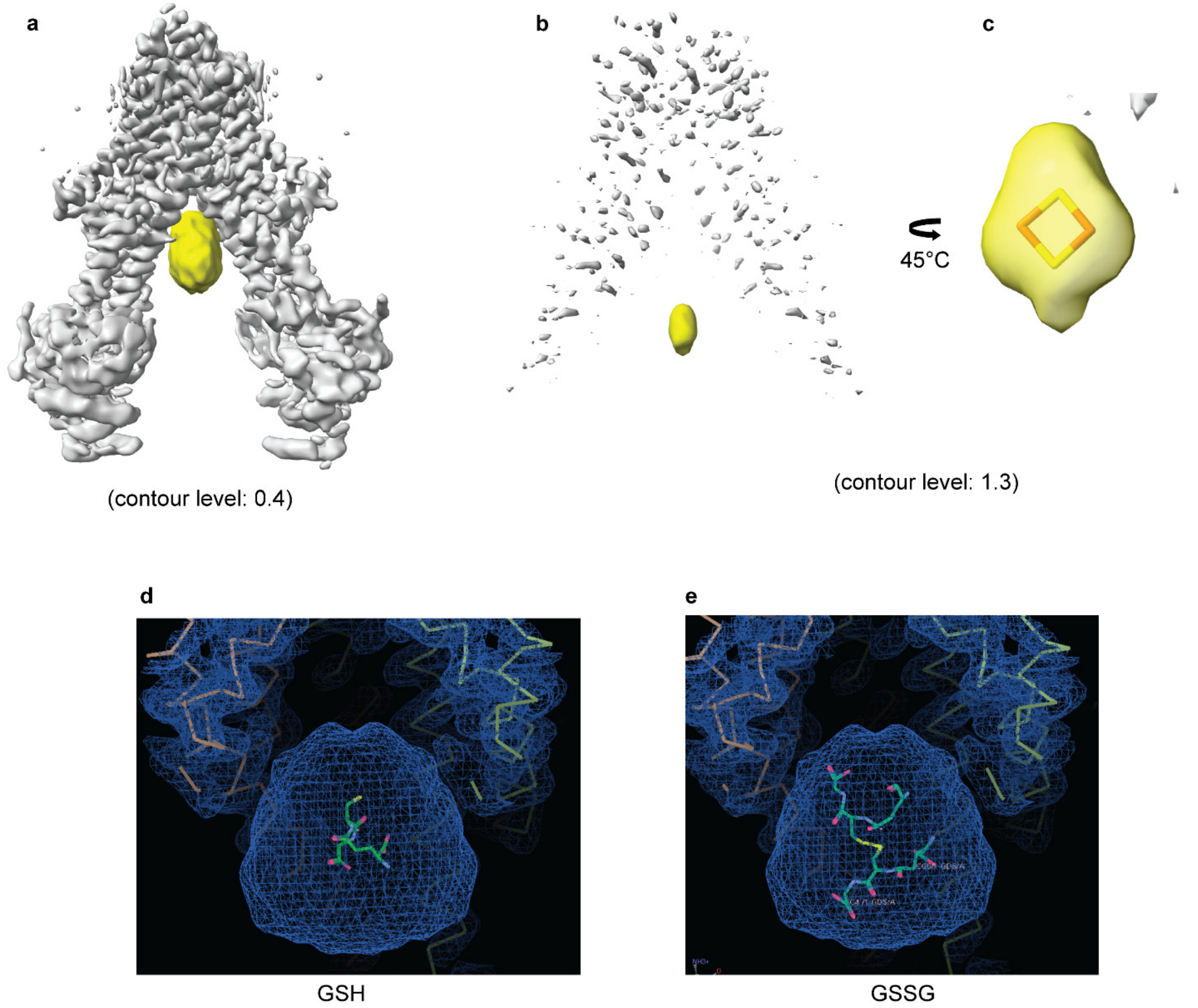

Supplementary Fig. 12: Cryo-EM density of inward-open state with bound cluster ($CtAtm1^{inw\text{-}opn/cluster}$). a Uncropped cryo-EM map at contour level 0.4 with CtAtm1 shown in grey, and the plate shape cluster density with volume of 3546 $Å^3$ is shown in yellow. **b** Map at contour level 1.3 with cluster density volume of 153 $Å^3$. **c** Close-view of panel b rotated 45° turn. [2Fe-2S] fits inside the cluster density. **d** GSH docking model to the cluster density. **e** GSSG docking model to the cluster density. See Fig. 2 for comparison to the cluster docking.

Supplementary Figure 13

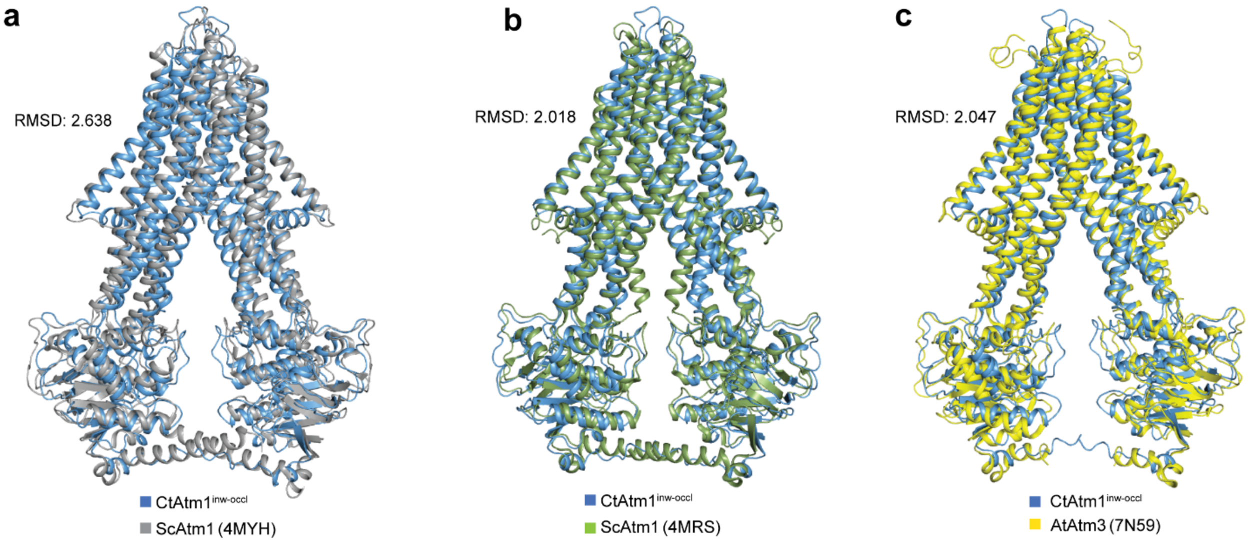

Supplementary Fig. 13: Structural comparison of cargo occluded CtAtm1, CtAtm1$^{\text{inw-opn-occl}}$, with ScAtm1, NaAtm1, and AtAtm3. a CtAtm1$^{\text{inw-opn-occl}}$ in blue is homologous to ScAtm1 (PDB-ID 4MYH) in grey with overall RMSD 2.6 Å. **b** CtAtm1$^{\text{inw-opn-occl}}$ in blue is homologous to NaAtm1 (PDB-ID 4MRS) in green with overall RMSD 2.0 Å. **c** CtAtm1$^{\text{inw-opn-occl}}$ in blue is homologous to AtAtm3 (PDB-ID 7N59) in yellow with overall RMSD 2.1 Å.

Supplementary Figure 14

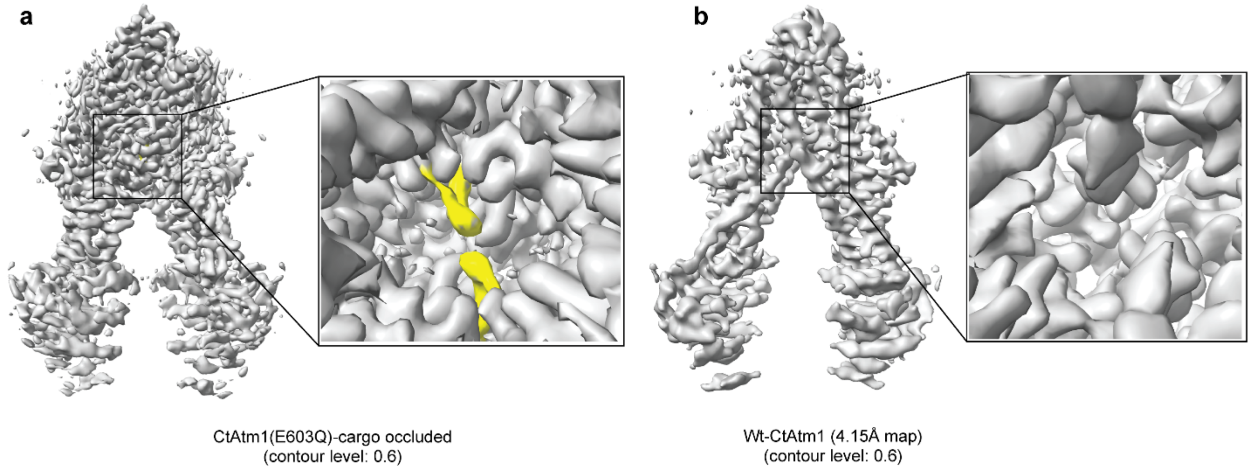

Supplementary Fig. 14: Structural comparison of cryo-EM densities in the two separate CtAtm1$^{inw\text{-}opn\text{-}occl}$ conformations, obtained from E603Q and wild-type sample, respectively.

Supplementary Figure 15

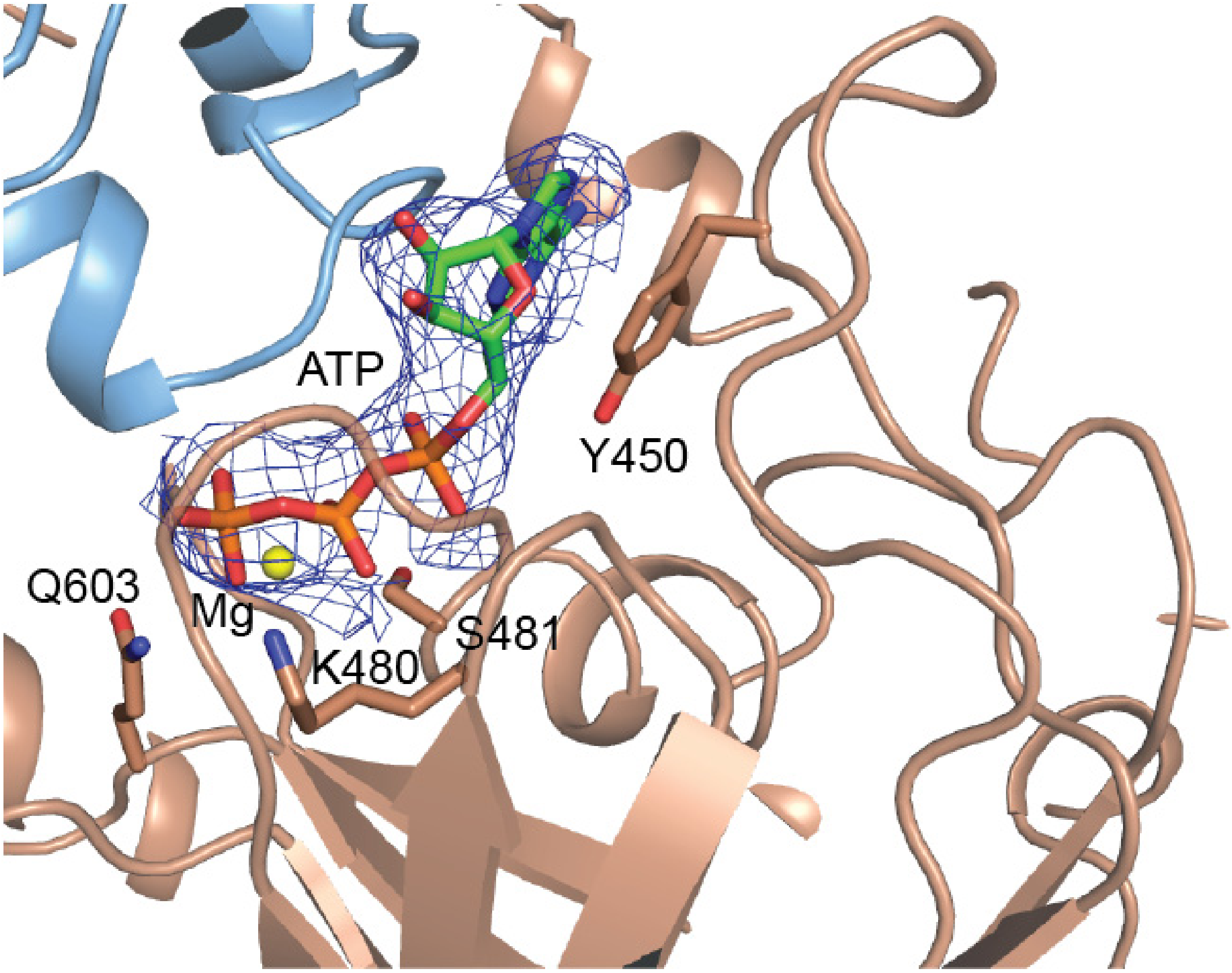

Supplementary Fig. 15. The nucleotide binding site of the CtAtm1$^{occl/ATP}$ structure. The blue mesh represents cryo-EM density at σ=7.0. ATP and residues are labeled and shown as sticks. Mg^{2+} is labeled and shown as a yellow sphere.

Supplementary Figure 16

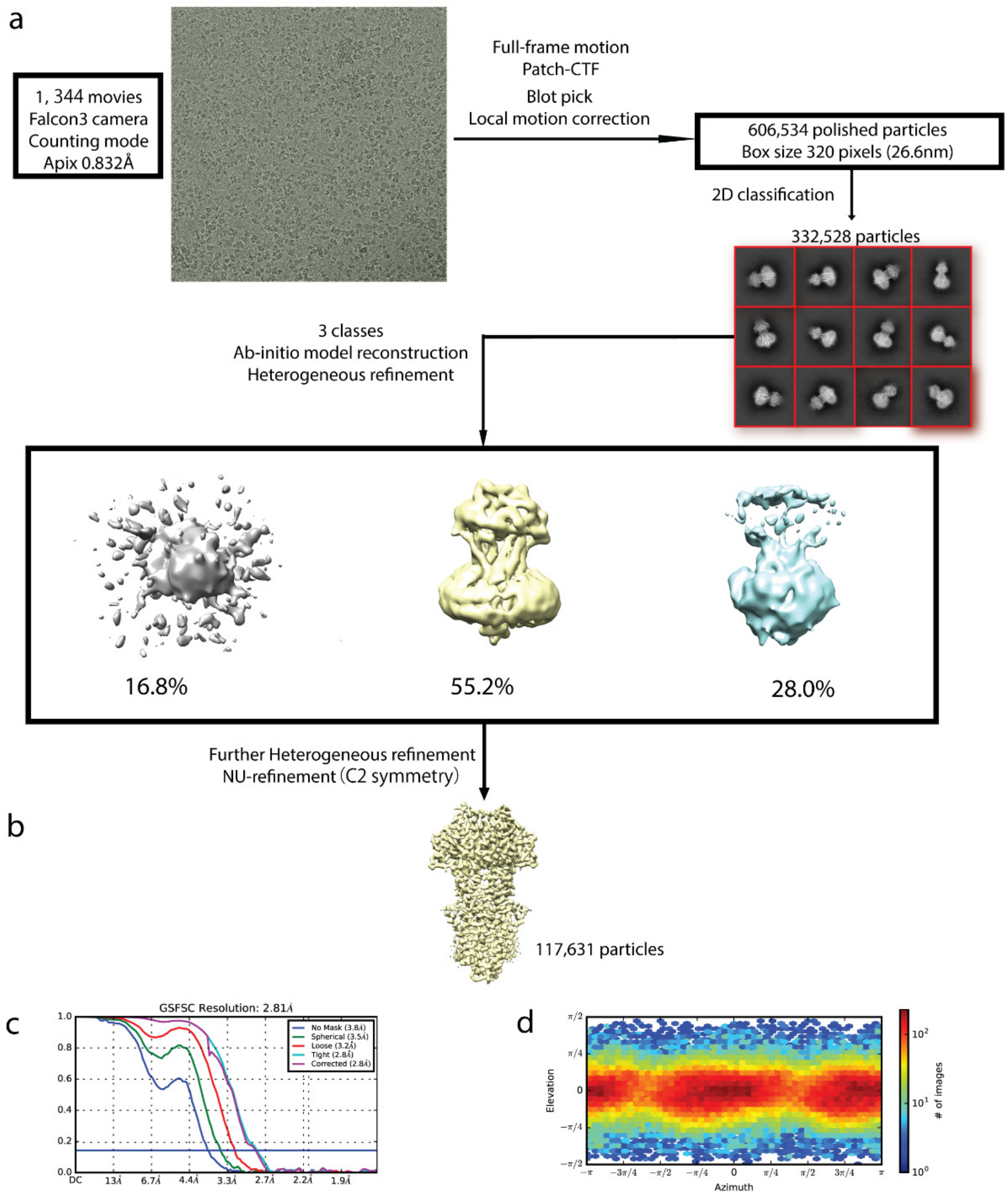

Supplementary Fig. 16: Representative micrograph and data processing of the CtAtm1 occluded state. a Representative micrograph and data processing flowchart. Please see the Methods section and Table S1 for details. **b** Reconstituted 3D cryo-EM maps. **c** Gold standard Fourier shell correlation (FSC) curve of the final map. **d** Particle orientation distributions in the final 3D reconstruction.

Supplementary Figure 17

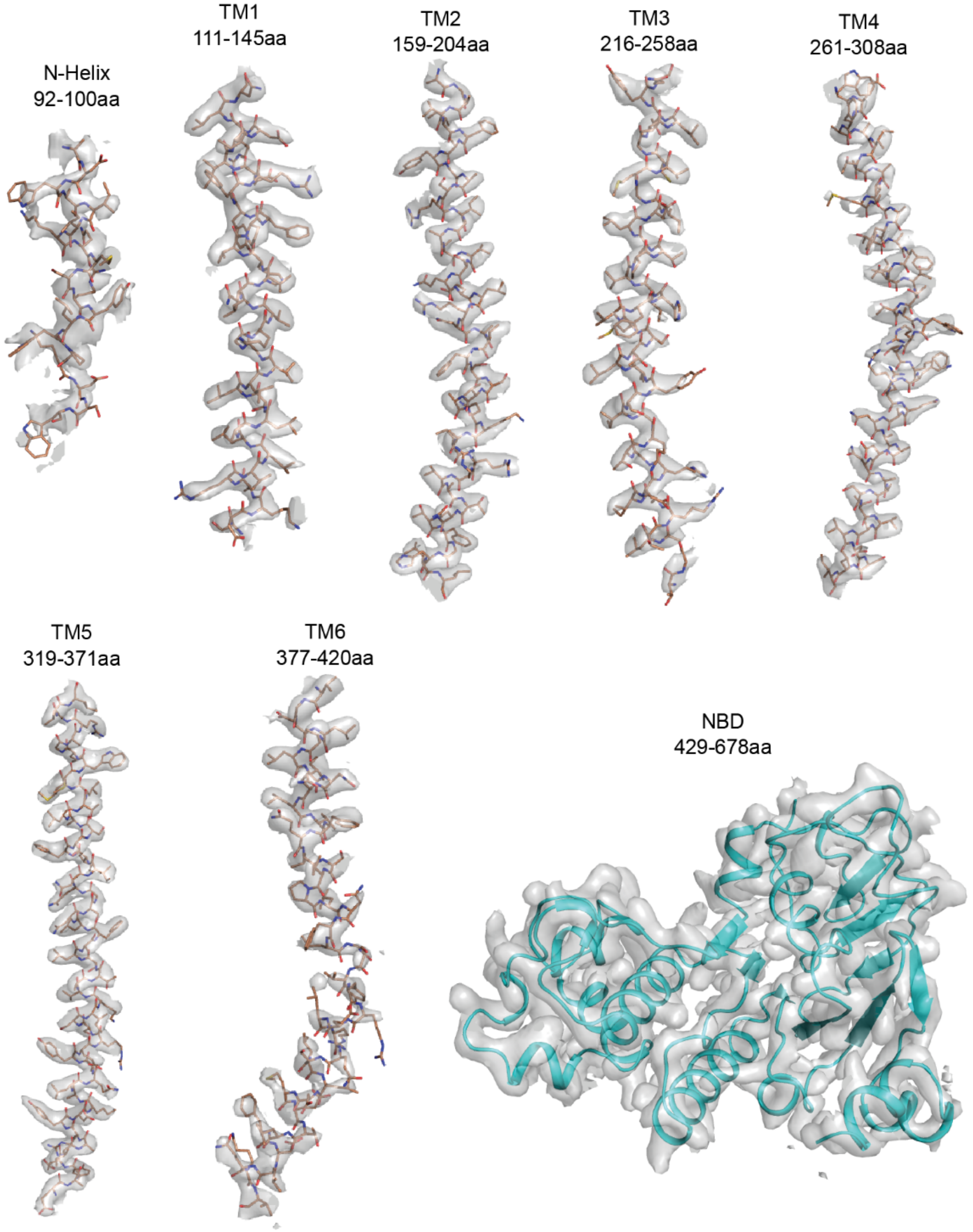

Supplementary Fig. 17 Quality of the Cryo-EM density in the occluded CtAtm1 state, CtAtm1$^{occl/ATP}$. Amino acid residue ranges are indicated and the map contour level is at σ=7.0, except for the N-terminal helix which is shown at σ=5.0.

Supplementary Figure 18

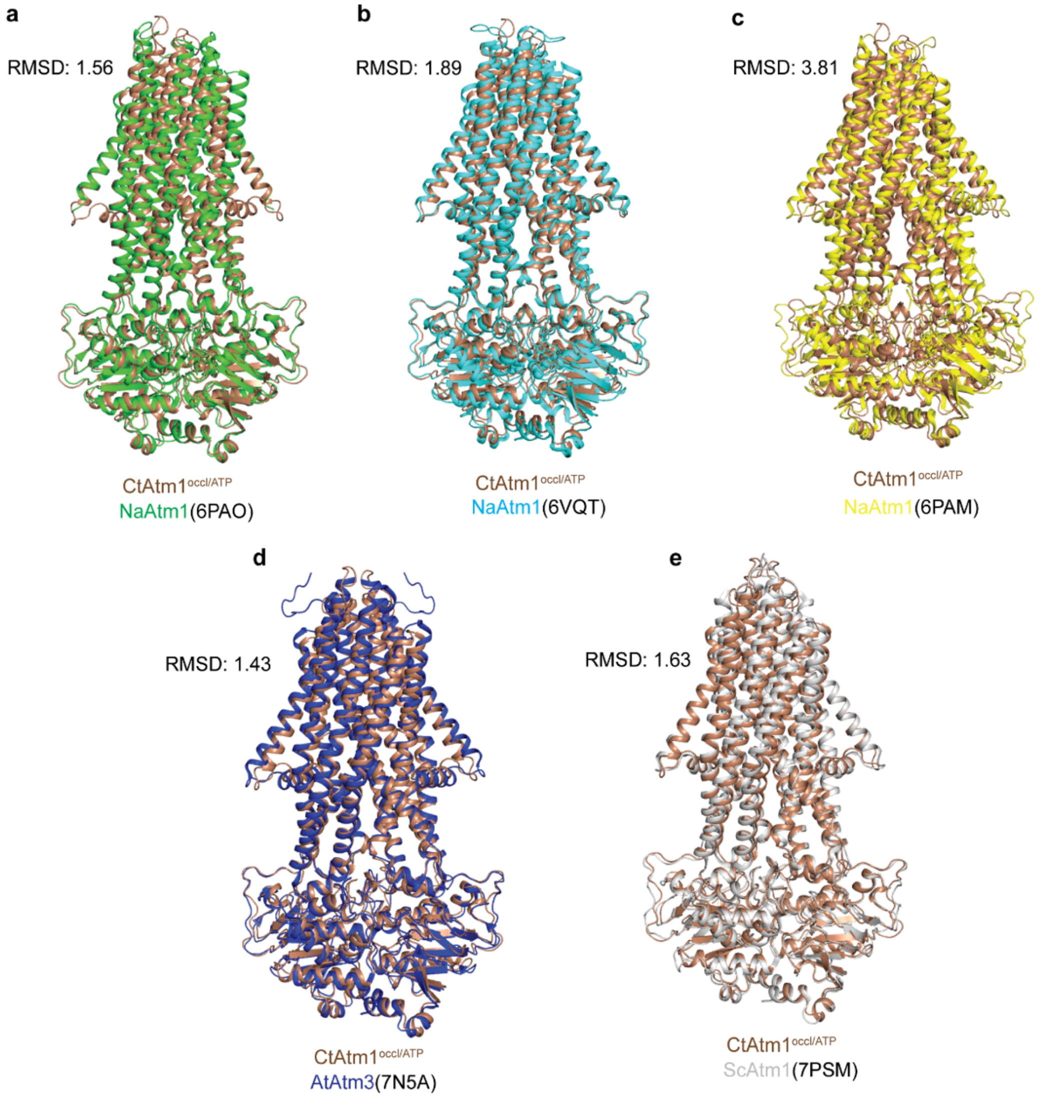

Supplementary Fig. 18: Structural comparison of occluded CtAtm1 with different occluded states of NaAtm1, AtAtm3 and ScAtm1. **a** Structural alignment of occluded CtAtm1 (brown) with occluded NaAtm1 (green, PDB-ID 6PAO) with overall RMSD 1.56 Å. **b** Structural alignment of occluded CtAtm1 (brown) with occluded NaAtm1 (cyan, PDB-ID 6VQT) with overall RMSD 1.89 Å. **c** Structural alignment of occluded CtAtm1 (brown) with occluded NaAtm1 (yellow, PDB-ID 6PAM) with overall RMSD 3.81 Å. **d** Structural alignment of occluded CtAtm1 (brown) with occluded AtAtm3 (blue, PDB-ID 7N5A) with overall RMSD 1.43 Å. **e** Structural alignment of occluded CtAtm1 (brown) with occluded ScAtm1 (grey, PDB-ID 7PSM) with overall RMSD 1.63 Å.

Supplementary Figure 19

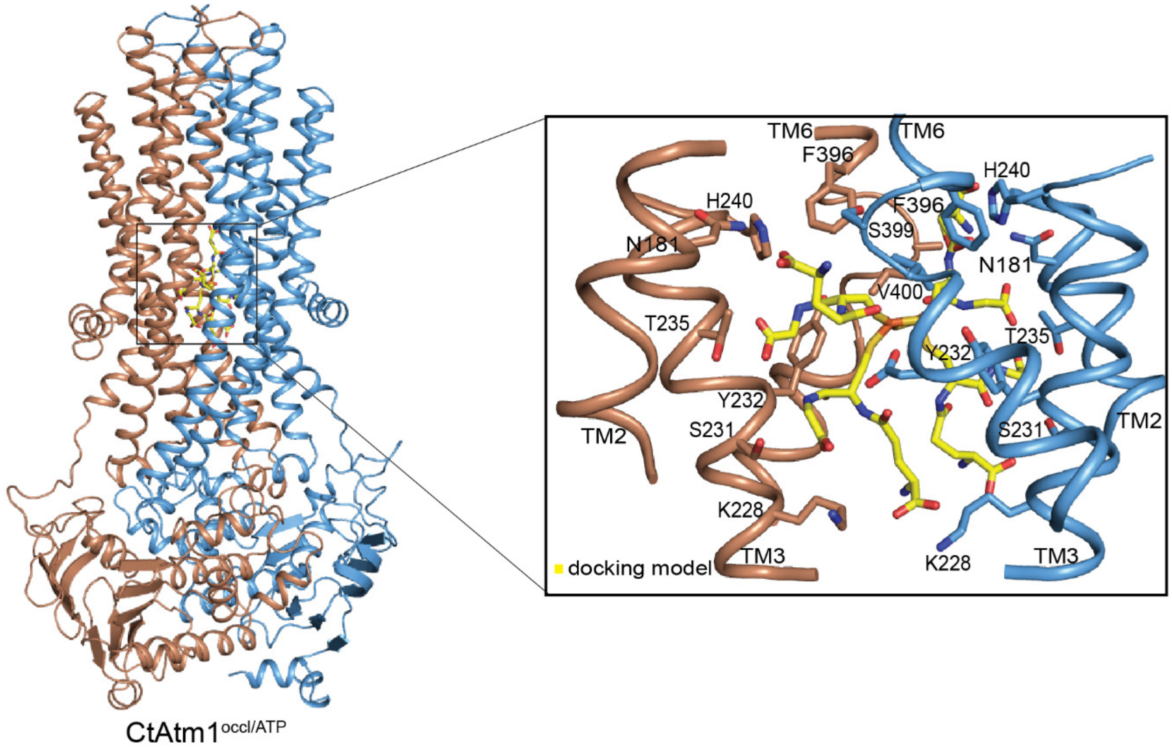

Supplementary Fig. 19: Docking model of the cluster to CtAtm1$^{occl/ATP}$. The cluster is shown in yellow and residues within 3 Å of the cluster, N181 of TM2, K228, S231, Y232, T235, H240 of TM3, D224, S231, Y232, T235, S236, H240 of TM3, and S399, V400 of TM6, are shown as sticks.

Supplementary Figure 20

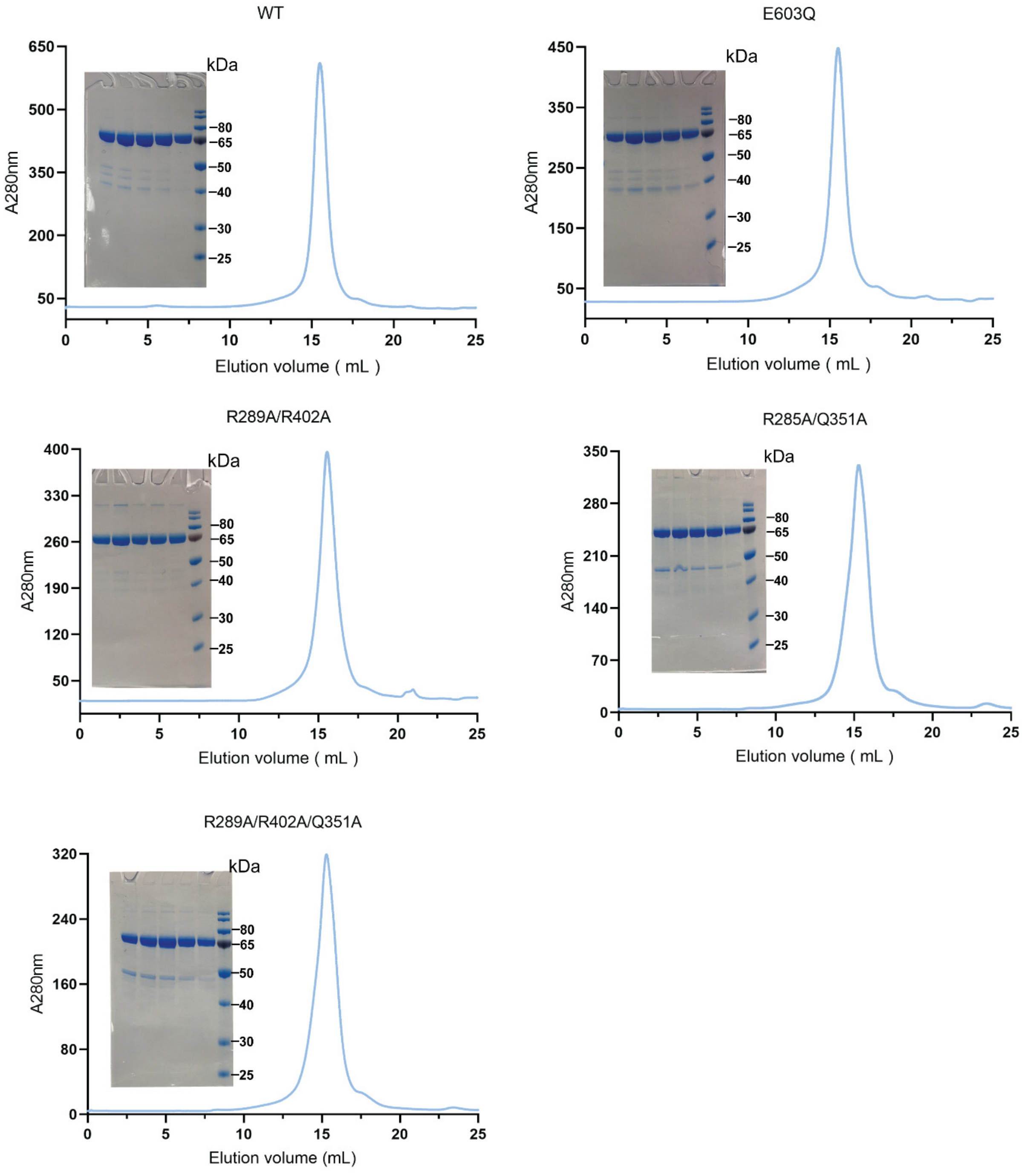

Supplementary Fig. 20: Size-exclusion chromatography profiles and SDS-PAGE analysis of employed CtAtm1 forms. The WT and E603Q overproduction and purification were performed at least five independent times, and the other mutants were isolated twice (independently).

Supplementary Figure 21

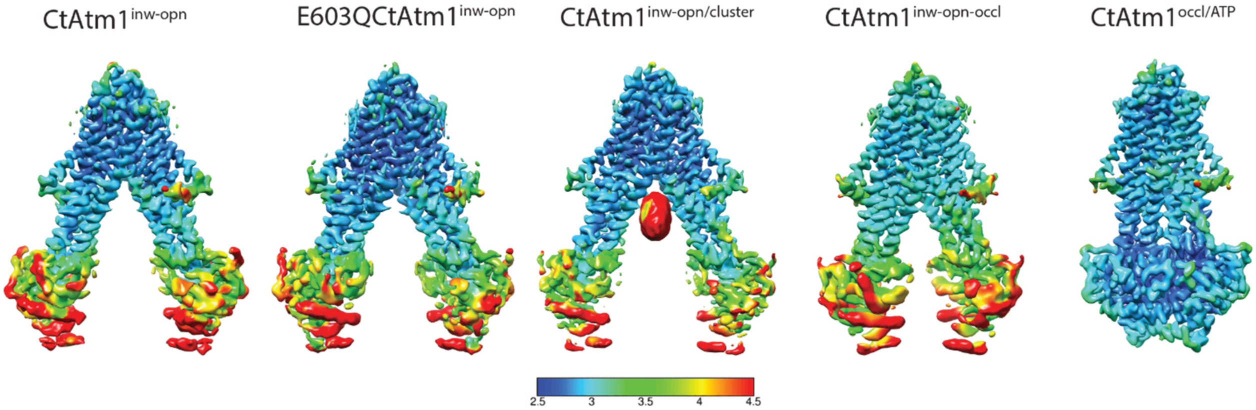

Supplementary Fig. 21: Local resolution estimation of the cryo-EM maps of the different generated states of CtAtm1.

Supplementary Table 1

	wild-type<br>Apo<br>CtAtm1$^{inw-opn}$	E603Q<br>Apo<br>E603QCtAtm1$^{inw-opn}$	E603Q<br>Cluster bound<br>CtAtm1$^{inw-opn/cluster}$	E603Q<br>Cargo bound<br>CtAtm1$^{inw-opn-occl}$	E603Q<br>ATP bound<br>CtAtm1$^{occl/ATP}$
	**PDB:7PQX**<br>**EMD-13606**	**PDB: 7PSD**<br>**EMD-13612**	**PDB: 7PRO**<br>**EMD-13609**	**PDB:7PRU**<br>**EMD-13610**	**PDB: 7PR1**<br>**EMD_13607**
**Data collection**					
EM equipment	FEI Titan Krios	FEI Titan Krios	FEI Titan Krios	FEI Titan Krios	FEI TitaKrios
Voltage (kV)	300	300	300	300	300
Detector	Falcon 3	Falcon3	Falcon 3	Falcon 3	Falcon 3
Data collection mode	counting	counting	counting	counting	counting
Pixle size (Å)	0.832	0.832	0.832	0.832	0.832
Energy filter	/	/	/	/	/
Electron dose ($e^-/Å^2$)	40	40	40	40	40
Defocus range (mm)	-1.2 ~ -2.6	-1.2 ~ -2.6	-1.2 ~ -2.6	-1.2 ~ -2.6	-1.2 ~ -2.6
**Data processing**					
Software	cryosparc	cryosparc	cryosparc	cryosparc	cryosparc
Number of final used particles	120619	108820	81145	60113	117631
Symmetry	C2	C2	C2	C2	C2
Map resolution (Å)	3.08	2.96	2.93	3.18	2.81
**Model refinement statistics**					
Total built residues	1160	1160	1160	1164	1174
Model-map-fit CC	0.84	0.88	0.84	0.83	0.83
**R.m.s.d.**					
bonds (Å)	0.008	0.015	0.012	0.009	0.006
angles (°)	0.918	1.107	1.083	1.027	0.857
**Molprobity statistics**					
Molprobity score	1.88	1.80	1.72	1.95	1.46
**Ramachandran plot**					

Favored (%)	94.72	94.46	95.16	93.19	97.86
Allowed (%)	5.28	5.54	4.84	6.81	2.14
Rotamer outliers (%)	0	0	0	0	0
Clash score	9.95	7.65	6.89	9.65	7.9
Average B-factor (Å^2)	70.03	76.33	72.49	97.99	76.28

Supplementary Table 1: Cryo-EM data collection and refinement statistics

Supplementary Table 2. Primers for cloning of CtAtm1.

Primers (Forward/Reverse)	
CtATM1-F	CATGCCATGGCAATGTCCCCTCGGTCCCGCCTCCTA
CtATM1-R	AGCGAGCTCTTTCTTCTCCTCCGCCTTCTCTCCCT
E603Q-F	TGTTCTTTGATCAGGCCACAAGTGC
E603Q-R	TTGTGGCCTGATCAAAGAACAGCAG
R289A-F	ACA AAG TTT GCA CGG CAG GCT A
R289A-R	CTGCCGTGCAAACTTTGTCCTC
R402A-F	GA TCA GTT TAC GCC GAA CTG C
R402A-R	CAGTTCGGCGTAAACTGATCCTAG
R285A-F	ACG GCA TGG GCG ACA AAG TTT AG
R285A-R	TTGTCGCCCATGCCGTAGTTGT
Q351A-F	AAC TCC GGG GCG AAC ATC ATC TTC
Q351A-R	GATGTTCGCCCCGGAGTTGAGGA

Uncropped SDS-PAGE gels used in Supplementary Fig. 20.

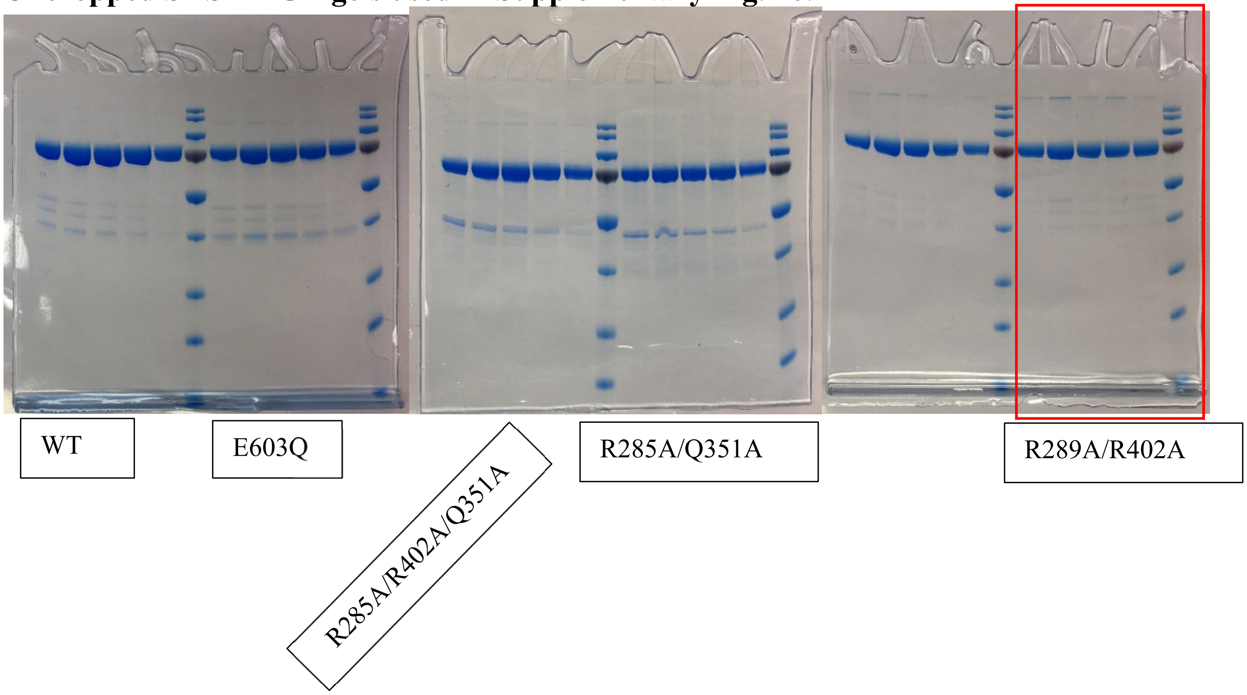

Supplement: Supplementary file 1 — Supplementary Information [file 41467_2022_32006_MOESM1_ESM.pdf]
