# Supplementary material for: Structures of Atm1 provide insight into [2Fe-2S] cluster export from mitochondria
Source: Nat Commun. 2022 Jul 27;13:4339. doi: 10.1038/s41467-022-32006-8 (PMC9329353; doi:10.1038/s41467-022-32006-8)
Supplement: Supplementary file 3 — Reporting Summary [file 41467_2022_32006_MOESM3_ESM.pdf]

nature research

Corresponding author(s): Pontus Gourdon

Last updated by author(s): Jun 28, 2022

Reporting Summary

Nature Research wishes to improve the reproducibility of the work that we publish. This form provides structure for consistency and transparency in reporting. For further information on Nature Research policies, see our Editorial Policies and the Editorial Policy Checklist.

Statistics

For all statistical analyses, confirm that the following items are present in the figure legend, table legend, main text, or Methods section.

n/a	Confirmed	
☐	☒	The exact sample size (n) for each experimental group/condition, given as a discrete number and unit of measurement
☐	☒	A statement on whether measurements were taken from distinct samples or whether the same sample was measured repeatedly
☒	☐	The statistical test(s) used AND whether they are one- or two-sided<br>*Only common tests should be described solely by name; describe more complex techniques in the Methods section.*
☒	☐	A description of all covariates tested
☒	☐	A description of any assumptions or corrections, such as tests of normality and adjustment for multiple comparisons
☐	☒	A full description of the statistical parameters including central tendency (e.g. means) or other basic estimates (e.g. regression coefficient) AND variation (e.g. standard deviation) or associated estimates of uncertainty (e.g. confidence intervals)
☒	☐	For null hypothesis testing, the test statistic (e.g. F, t, r) with confidence intervals, effect sizes, degrees of freedom and P value noted<br>*Give P values as exact values whenever suitable.*
☒	☐	For Bayesian analysis, information on the choice of priors and Markov chain Monte Carlo settings
☒	☐	For hierarchical and complex designs, identification of the appropriate level for tests and full reporting of outcomes
☒	☐	Estimates of effect sizes (e.g. Cohen's d, Pearson's r), indicating how they were calculated

Our web collection on statistics for biologists contains articles on many of the points above.

Software and code

Policy information about availability of computer code

Data collection	Single particle cryo-EM data were collected on Titan Krios electron microscopes with a Falcon3 detector
Data analysis	Cryo-EM data were processed with cryosparc v 2.15.0(https://cryosparc.com). Model building was performed using USCF chimera 1.14, Wincoot 0.9.2 , and phenix1.18.2. MolProbity (phenix1.18.2).The functional data was assessed using Graph Pad Prism 9 (version 9.3.0). Figures were generated using pymol 2.3.4 and ChimeraX version 1.2 (2020-10-07). Alignments were performed using Clustal Omega (https://www.ebi.ac.uk/Tools/msa/clustalo/), and visualized using ESPript 3.0(https://espript.ibcp.fr/ESPript/ESPript/).

For manuscripts utilizing custom algorithms or software that are central to the research but not yet described in published literature, software must be made available to editors and reviewers. We strongly encourage code deposition in a community repository (e.g. GitHub). See the Nature Research guidelines for submitting code & software for further information.

Data

Policy information about availability of data

All manuscripts must include a data availability statement. This statement should provide the following information, where applicable:

- Accession codes, unique identifiers, or web links for publicly available datasets
- A list of figures that have associated raw data
- A description of any restrictions on data availability

The structures and Cryo-EM maps have been deposited to the PDB with the following accession numbers: PDB-ID 7PQX/EMD13606-for CtAtm1-inw-opn, PDB-ID 7PSD/EMD-13612 for E603QCtAtm1-inw-opn, PDB-ID 7PRO/EMD-13609 for CtAtm1-inw-opn/cluster, PDB-ID 7PRU/EMD-13610 for CtAtm1-inw-opn-occl, and PDB-ID 7PR1/EMD-13607 for CtAtm1-occl/ATP. The raw data of the functional assay is provided in the source data file.

Field-specific reporting

Please select the one below that is the best fit for your research. If you are not sure, read the appropriate sections before making your selection.

- [x] Life sciences
- [ ] Behavioural & social sciences
- [ ] Ecological, evolutionary & environmental sciences

For a reference copy of the document with all sections, see nature.com/documents/nr-reporting-summary-flat.pdf

Life sciences study design

All studies must disclose on these points even when the disclosure is negative.

Sample size	Complete Cryo-EM statistics are provided in the Methods section, Supplementary Table 1 and Supplementary Figures 4, 9, 10 and 16. Each structure is determined from particles from one selected grid. Sample size determination was not used for the enzymatic assay. The sample size was selected based on the standard in the literature (three independent experiments).
Data exclusions	Complete Cryo-EM statistics are provided in the Methods section, Supplementary Table 1 and Supplementary Figures 4, 9, 10 and 16. No data were excluded for the biochemical assay.
Replication	The wild-type and E603Q full-length protein forms were produced and and purified multiple times and on different occasions and other mutants were produced and purified twice in different days independently. Nanodiscs reconstitution were performed multiple times independently. All purifications were successful. Multiple cryo-EM grids were prepared and frozen, data was collected for one selected grid for wild-type CtAtm1inw-opw state, one selected grid for CtAtm1occl/ATP and one selected grid for E603QCtAtm1in-opw, CtAtm1inw-opn/cluster and CtAtm1inw-opn-occl. CtAtm1 The activity assay without any cargo supplied was performed in two replicates of two biological independent purifications, the activity assay with cargo was performed by three independent measurements, and the ICP-MS was performed using three independent measurements, and all data are included in the analysis (no failed attempts).
Randomization	Extracted particles were randomly assigned to calculate gold-standard FSC. For the biochemical assay, the protein samples were assessed randomly, and all samples were treated in an identical manner.
Blinding	No blinding was applied as no group allocation was used.

Reporting for specific materials, systems and methods

We require information from authors about some types of materials, experimental systems and methods used in many studies. Here, indicate whether each material, system or method listed is relevant to your study. If you are not sure if a list item applies to your research, read the appropriate section before selecting a response.

Materials & experimental systems

n/a	Involved in the study
x	Antibodies
x	Eukaryotic cell lines
x	Palaeontology and archaeology
x	Animals and other organisms
x	Human research participants
x	Clinical data
x	Dual use research of concern

Methods

n/a	Involved in the study
x	ChIP-seq
x	Flow cytometry
x	MRI-based neuroimaging
